# Fast Quantitative Validation of 3D Models of Low-Affinity Protein–Ligand Complexes by STD NMR Spectroscopy

**DOI:** 10.1021/acs.jmedchem.4c00204

**Published:** 2024-06-07

**Authors:** Ridvan Nepravishta, Jonathan Ramírez-Cárdenas, Gabriel Rocha, Samuel Walpole, Thomas Hicks, Serena Monaco, Juan C. Muñoz-García, Jesús Angulo

**Affiliations:** † School of Pharmacy, University of East Anglia, Norwich Research Park, Norwich NR4 7TJ, U.K.; ‡ Institute for Chemical Research (IIQ), CSIC - University of Seville, 49 Américo Vespucio, 41092 Seville, Spain; § Cancer Research Horizons, CRUK Scotland Institute, Garscube Estate, Switchback Road, Bearsden, Glasgow G61 1BD, U.K.

## Abstract

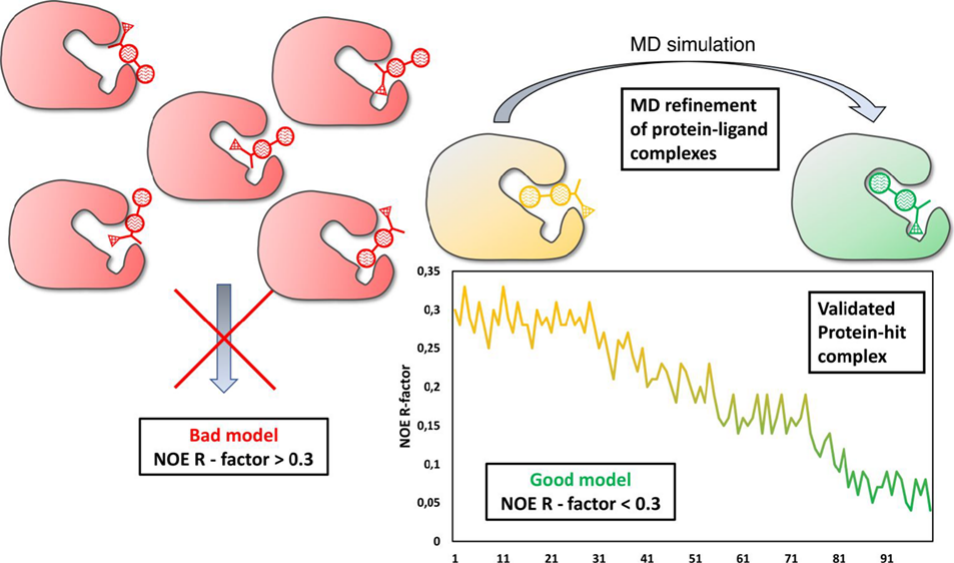

Low-affinity protein–ligand interactions are important for many biological processes, including cell communication, signal transduction, and immune responses. Structural characterization of these complexes is also critical for the development of new drugs through fragment-based drug discovery (FBDD), but it is challenging due to the low affinity of fragments for the binding site. Saturation transfer difference (STD) NMR spectroscopy has revolutionized the study of low-affinity receptor–ligand interactions enabling binding detection and structural characterization. Comparison of relaxation and exchange matrix calculations with ^1^H STD NMR experimental data is essential for the validation of 3D structures of protein–ligand complexes. In this work, we present a new approach based on the calculation of a reduced relaxation matrix, in combination with funnel metadynamics MD simulations, that allows a very fast generation of experimentally STD-NMR-validated 3D structures of low-affinity protein–ligand complexes.

## Introduction

Low-affinity protein–ligand interactions play a crucial role within the extensive framework of physical and functional protein interactions that support life across biological organisms. They serve as essential contributors to the regulation of biological processes, including cell–cell communication, signal transduction, immune response differentiation, and protein phase transitions.^1,2^ A significant example of prevalent and essential specific low-affinity interactions is seen in the case of protein–carbohydrate complexes. Evolution has provided cells with a highly glycosylated surface layer, known as the glycocalyx, which plays a critical role in cell–cell communication, adhesion, and cell–microorganism interactions by binding to various receptors.^3−5^ Additionally, a precise structural characterization of low-affinity protein–ligand complexes is essential for progressing fragments, targeting a protein of interest, during the optimization of hit-to-lead compounds through a rational design, as part of the fragment-based drug discovery (FBDD) process. Although these initial fragments are important starting points for the future development of potent drugs, they are characterized by very weak affinities (dissociation constants typically from micromolar to millimolar).^6,7^

Compared to tightly bound complexes, the determination of functionally relevant 3D structures of low-affinity protein–ligand complexes is significantly more challenging because they are much more sensitive to variations in their environment, due to their short residence time in the bound state. The three-dimensional structures of protein–ligand complexes obtained by techniques, such as X-ray diffraction or cryo-EM, have provided invaluable insights into the structural details of protein–ligand complexes over the years. However, the perturbation of the system caused by the requirements of the experimental technique used has raised some debate.^8−11^

In this sense, orthogonal techniques that might validate in solution-state models achieved either by X-ray or Cryo-EM are very important. Indeed, NMR spectroscopy has played a key role in the analysis of 3D structural determinants of specificity in the molecular recognition of small ligands by proteins in solution.^12−15^ Among the so-called ligand-observed NMR strategies, WaterLOGSY^16,17^ and STD NMR are powerful tools to probe and characterize low-affinity protein–ligand interactions,^18^ conveniently relying on the acquisition of ligand ^1^H NMR spectra in the presence of small amounts of the protein without the need for isotopic enrichment. STD NMR has been shown to be useful for analyzing the orientation, binding determinants, and ligand conformation within the complex by using 3D molecular models that best match the experimental data.^19−22^ In the STD NMR experiment, the transfer of saturation in the bound state to different ligand protons depends, broadly speaking, on their respective distances to the protons of the protein in the binding pocket, so that the intensities reflect the spatial contacts of the ligand with the protein. Classification of the STD intensities in relative terms among the ligand protons renders the map of close contacts of the ligand with the protein, called binding epitope mapping.

A recent development of STD NMR, called DEEP-STD NMR,^23,24^ has taken this concept further by determining differential epitope mappings. These allow the identification of the types of protein side chains (aromatic, aliphatic, polar, and apolar) that surround the ligand in the binding pocket. Together with a 3D model of the protein, differential epitope mappings facilitate the determination of the orientation of the ligand within the architecture of the binding site.

In general, binding epitope mappings should not be obtained using a single saturation time, as errors may arise due to (i) differences in ligand T_1_ relaxation between protons, (ii) binding kinetics, (iii) fast rebinding effects, and (iv) extent of saturation. To minimize the effects of these factors, Mayer and James proposed to acquire a series of STD NMR experiments at increasing saturation times (build-up curves). In this procedure, the binding epitopes are obtained from the analysis of the initial slopes by fitting the experimental STD data points to the monoexponential equation:^25^
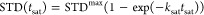
1where STD(*t*_sat_) is the STD factor of a given resonance obtained as *I*_0_ – *I*_sat_/*I*_0_ at a saturation time *t*_sat_, STD^max^ is the maximum asymptotic value obtained from the fit, *k*_sat_ is the saturation rate constant, and *t*_sat_ is the saturation time. The initial slope, STD_0_, is obtained from eq 2:
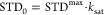
2

The STD_0_ values are then normalized to the highest value within the set of ligand protons.^26^

Binding epitope mappings from STD NMR studies can inform subsequent optimization of identified hits in FBDD, but arguably the best aid to the medicinal chemist comes from the availability of experimentally validated 3D molecular models of the protein–ligand complexes. In cases where crystallization of the complexes is unsuccessful, the process of ligand optimization (“hit-to-lead”) will greatly benefit from the availability of experimentally validated 3D molecular models by STD NMR. Furthermore, it would be desirable to use STD NMR spectroscopy to validate 3D dynamic ensembles of protein–ligand complexes in solution by confronting the experimental data with the resulting trajectories from molecular dynamics (MD) simulations.

Previous attempts to score sets of 3D molecular models of protein–ligand complexes, based on deviations from experimental binding epitope mappings, have used algorithms based on protein–ligand distance hierarchies.^27,28^ However, purely distance-based algorithms suffer from a lack of description of the dipole–dipole relaxation processes, so that the effect of internal motions in cross-relaxation (NOE) is completely absent. This may significantly affect the analysis of protein–ligand complexes with substantial internal dynamics in the binding pocket.

Full relaxation matrix approaches have also been previously applied to validate static and dynamic models of protein–ligand complexes by STD NMR.^29^ However, such approaches are time-consuming, making them impractical for analyzing very long MD simulations and simultaneously deriving theoretical full-STD build-up curves for each frame of the simulation. To reduce the computational burden, the application of these full-matrix approaches has typically been limited to the use of a “single saturation time” analysis of MD trajectories.^30^ This type of analysis might be prone to false optimal structures when scoring is based on a best-fitting factor value (NOE R-factor), as the full-matrix calculation does not predict the entire saturation build-up. Thus, a given protein–ligand complex might yield a poor (high) NOE R-factor when considering the entire STD build-up curve, while a relatively good (low) NOE R-factor might be obtained when using a single saturation time, hence leading to an incorrect model validation.

In this paper, we present a reduced matrix theoretical approach to predict accurate and fast STD NMR initial slopes from 3D molecular models of low-affinity protein–ligand complexes. The novel approach fully accounts for both the network of dipole–dipole couplings and the relaxation processes present at the protein–ligand interface. Once the binding site is characterized (e.g., through competition studies or using paramagnetic relaxation enhancement (PRE) probes in solution),^31^ this allows to (i) perform very fast calculations of theoretical STD initial slopes (STD_0_^cal^) from a 3D molecular model or an MD trajectory of the protein–ligand complex and (ii) explicitly include experimental STD initial slopes (STD_0_^exp^) from the complete STD build-up curves for the validation of receptor–ligand 3D structures in solution.

### Development of the Reduced Matrix Approach

**Scheme 1:**
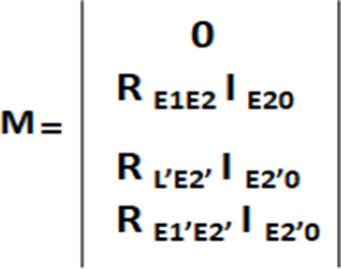
**M** Matrix Definition

The complete relaxation matrix for a two-site exchange system has already been described by Jayalakshmi et al.^32^ In this model, protein protons are divided into (i) E1 and E1′, protein protons not directly affected by saturation, in the free and bound states, respectively, and (ii) E2 and E2′, protein protons directly affected by saturation, in the free and bound states, respectively. The theoretical STD NMR build-up curves are then derived using the following equation.
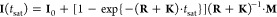
3where **I**_0_ is a matrix containing the intensities of the ligand protons at thermal equilibrium, **I**(*t*_sat_) is the matrix of intensities of the ligand protons after protein saturation, **R** is the general relaxation rate matrix for the ligand, **K** is the generalized exchange kinetics matrix, and **M** is a general matrix term containing elements of both protein and ligand species in their bound and free forms. Equation 3 is similar to the monoexponential equation proposed by Mayer and James for the fitting of experimental STD NMR data (eq 1).^25^ By comparing the equations describing the experimental (eq 1) and theoretical (eq 3) STD NMR build-up curves, we notice that the term (**R** + **K**) in the exponential function bears resemblance to the saturation rate constant, *k*_sat_ (acting as a matrix of *k*_sat_ values for each ligand proton), while the term (**R** + **K**)^−1^·**M** can be likened to a matrix of STD^max^ values. Based on these observations, eq 2 can be rearranged to derive (STD_0_^cal^) as
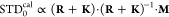
4

which simplifies to
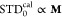
5where STD_0_^cal^ is a matrix containing the calculated initial slopes for each ligand proton, and **M** is a matrix defined as in Scheme 1.

In Scheme 1, **R**_**E1E2**_ is the cross-relaxation matrix between the unsaturated (E1) and directly saturated (E2) protein protons in the free state multiplied by the thermal equilibrium intensity of the directly saturated protein protons (E2) in the free state **I**_**E20**_; **R**_**L’E2’**_ is the cross-relaxation matrix between the ligand protons in the bound state (L1’) and the directly saturated protein protons in the bound state (E2’) multiplied by the thermal equilibrium intensity of the saturated bound protons (E2’) **I**_**E2’0**_; **R**_**E1’E2’**_ is the cross-relaxation matrix between the unsaturated and directly saturated protein protons in the bound state (E1’ and E2’) multiplied by the thermal equilibrium intensity of the directly saturated protein protons in the bound state (E2’) **I**_**E2’0**_. The calculation of submatrix **M** hence allows for the direct determination of a matrix containing all the individual STD initial slopes (STD_0_^cal^). It should be noted this is remakably different from the full-relaxation matrix approach used by CORCEMA-ST, which involves the calculation of all exchange kinetics (only truly meaningful over extended time frames) and relaxation rate matrices for all saturation times of a full STD NMR build-up curve. On the contrary, our reduced matrix approach simply implies the calculation of cross-relaxation and thermal equilibrium intensity terms (see Scheme 1). This leads to two main advantages, i.e. (i) the great simplification of theoretical initial slope calculation, hence boosting the computational speed and (ii) the significant reduction of calculation dependence on many parameters requiring previous experimental knowledge or being subjected to rounds of optimization (e.g., kinetics of binding). We have called this matrix “Reduced” because not all matrixes are considered in the calculation but only a subset of them.

Our new approach for fast quantitative validation of 3D models of low-affinity protein–ligand complexes by STD NMR data is based on the determination of the reduced relaxation matrix **M** (eq 5; Scheme 1). We have implemented the protocol in the form of a web application, called *RedMat*, which takes as input data (i) the Cartesian coordinates of the protein–ligand complex, (ii) the experimental STD initial slopes (STD_0_ values), in relative scale, i.e., normalized to the highest intensity STD_0_ (arbitrarily assigned a value of 100%), (iii) the protein protons initially saturated in the STD NMR experiment, and (iv) a number of additional experimental parameters. These parameters include: the NMR spectrometer frequency (in MHz), the rotational correlation time of the bound protein–ligand complex (in ns), the concentrations of ligand and protein (both in μM units), the dissociation constant of the protein ligand complex (in μM units), and the cutoff distance (in Å units) for protein protons from the ligand to be considered in the calculation.

*RedMat* can process static 3D structures of complexes as well as dynamic ensembles from molecular dynamics (MD) simulations, in a time-efficient manner. The docking mode accepts coordinate sets in the form of Protein Data Bank (PDB) files, one for the protein and one for the ligand. Predicted initial slope values (STD_0_^cal,*k*^) are output, and their agreement with experimental STD_0_ factors is evaluated using the NOE R-factor (eq 6) for each ligand proton *k*, defined as
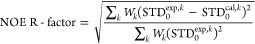
6where *W*_*k*_ is a weighting factor (in our NOE R-factor calculation *W*_*k*_ = 1), STD_0_^exp, *k*^ is the experimental STD_0_ value obtained for the *k*-th proton of the ligand, while STD_0_^cal,*k*^ is the calculated STD_0_ value using the reduced matrix approach presented in this paper. As a rule of thumb, an NOE R-factor lower than 0.3 indicates a good agreement between the calculated and experimental STD_0_ values. If multiple 3D models are present in the ligand file (i.e., different ligand poses from docking calculations), separate calculations are performed for each model, making this mode useful for quantitatively assessing the agreement of a set of molecular docking binding poses with the experimental STD NMR data. The dynamic mode pinpoints the excellent time-efficient character of our approach, allowing MD trajectories to be used as input files. It accepts AMBER^33^ topology and trajectory files, providing the evolution of the NOE R-factor as a function of the simulation time. This type of analysis is useful for elucidating: (i) the extent to which changes in ligand conformation and/or orientation along the MD simulation affect the agreement with the experimental data and (ii) which conformational populations from the entire dynamic ensemble are in best agreement with the experimental NMR data. In addition, *RedMat* allows individual frames to be downloaded for further analysis. For flexible receptors and ligands, the dynamic mode significantly improves the analysis over static models from rigid or semirigid docking calculations, which do not fully account for the dynamics of both molecular partners.

## Results and Discussion

To test this novel approach, we studied five low-affinity protein–ligand complexes (PDB codes: 4X4A, 5M3A, 5M39, 6MSY, 6GH2) for which high-resolution X-ray and STD_0_ values were available.^23,34,35^ We describe here the results for three complexes (4X4A, 5M3A, and 5M39), while the other two (6MSY and 6GH2) are shown in the ESI. In all cases, we ran both application modes, first, by calculating the reduced relaxation matrix from the experimentally determined X-ray crystal structures of the protein–ligand complexes, and second, by performing the calculation on 100 ns unbiased MD trajectories as well as funnel metadynamics (funnel-MD) simulations. This last technique was preferred because during the funnel-MD simulation, the new binding poses generated are independent from the starting point, which is very important for those cases where a crystallographic structure of the complex does not exist. The STD_0_ calculation of all MD trajectories was performed assuming that all protein methyl protons are saturated, and a cutoff distance of 10–12 Å from the ligand (for complexes 5M3A, 5M39, 4X4A) was considered. This allows the inclusion of all protein protons that contribute most to the saturation transfer at the protein–ligand interface in the STD NMR experiment.

### Gut Intramolecular Trans-Sialidase RgNanH-GH33-2,7-anhydro-Neu5Ac Complex

#### Validation of Static 3D Models

**Figure 1:**
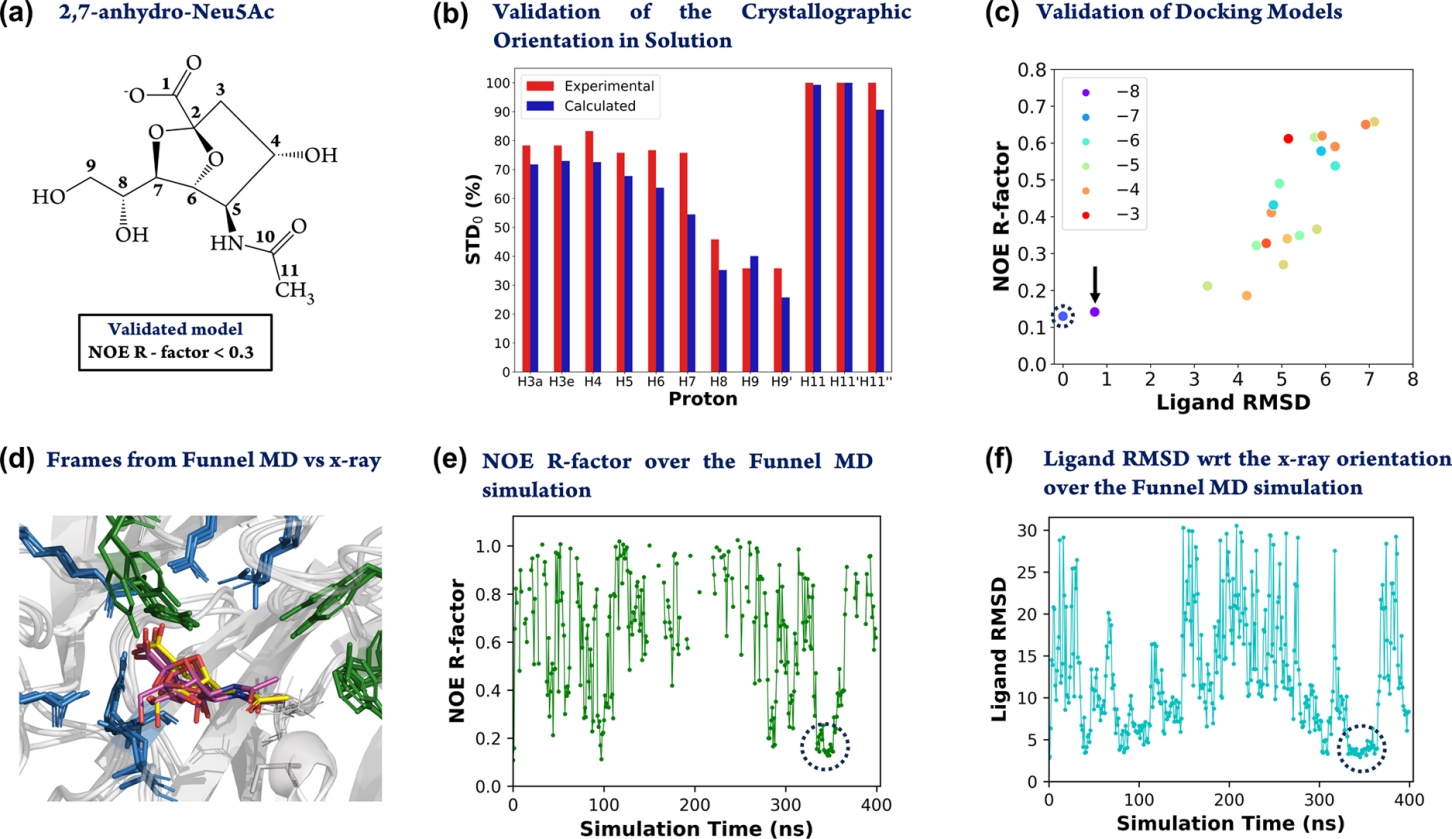
*RedMat* analysis of 2,7-anhydro-Neu5Ac binding to *Rg*NanH-GH33 (PDB code 4X4A). (a) Two-dimensional sketch of the structure of the 2,7-anhydro-Neu5Ac ligand. The labels associated with the ligand proton are shown next to it. (b) Comparison between the calculated (from the X-ray structure; red bars) and experimental (blue bars) relative STD_0_ factors (binding epitope mapping) of the nonexchangeable protons of 2,7-anhydro-Neu5Ac binding to *Rg*NanH-GH33. A NOE R-factor of 0.13 was obtained. (c) Two-dimensional plot representing the NOE R-factor as a function of the ligand RMSD for the docking poses obtained for 2,7-anhydro-Neu5Ac binding to *Rg*NanH-GH33. The docking score of each pose is indicated by the color code shown in the legend. The data point corresponding to the X-ray structure is highlighted with a dashed circle. (d) Superposition of three frames of the funnel-MD simulation (protein in light gray cartoon, and ligand in magenta sticks) and the X-ray structure (protein in gray colored cartoon, and ligand in yellow sticks) of the complex. The protein residues within 12 Å from the ligand, shown as wheat-colored lines, were included in the calculation of theoretical STD_0_ values. (e) Evolution of the NOE R-factor of 2,7-anhydro-Neu5Ac over 400 ns of funnel-MD simulation. The fragment of the trajectory where the ligand adopts an X-ray-type of orientation is highlighted with a dashed circle and corresponds with the lower ligand RMSD region shown in (f). Unconnected data points indicate MD simulation frames when the ligand is dissociated from the protein and, hence, no NOE R-factor is calculated. (f) Evolution of the root-mean-square deviation (RMSD) for the 2,7-anhydro-Neu5Ac ligand (all atoms except the protons considered) with respect to the protein-binding site (residues within 6 Å from the ligand considered). The fragment of the trajectory where the ligand adopts an X-ray-type of orientation is highlighted with a dashed circle.

The first system studied was the complex of the catalytic domain of the intramolecular trans-sialidase from *Ruminococcus gnavusRg*NanH-GH33 with the ligand 2,7-anhydro-Neu5Ac (PDB code: 4X4A).^36^ This system has been thoroughly characterized before by STD NMR in our research group due to its high biological relevance.^23^ In particular, the elucidation of the binding determinants governing the interaction of 2,7-anhydro-Neu5Ac (Figure 1a) to sialidases is of great interest for understanding the mechanisms of gut microbiota adaptation. For *RedMat* calculations, we used a rotational correlation time of the protein of 34.5 ns, estimated with HYDRONMR,^37^ and a dissociation constant of 2000 μM. The protein and ligand concentrations were 20 and 1000 μM, respectively, according to the experimental conditions.^23^

Figure 1b shows the comparison of the experimental binding epitope (red bars) with the theoretical epitope (blue bars) of the 2,7-anhydro-Neu5Ac ligand obtained using *RedMat* for the X-ray structure 4X4A. The NOE R-factor of the X-ray structure was 0.13 using a cutoff of 12 Å, indicating a very good fit with the STD NMR binding epitope. In addition, we tested the robustness of RedMat for the identification of docking poses in agreement with the experimental STD NMR binding epitope (Figure 1c) and with an existing X-ray structure. Figure 1c highlights with a black arrow the docking pose resembling the X-ray orientation (out of the 20 clusters obtained from docking). Clearly, the pose is characterized by a low NOE R-factor, a low ligand RMSD (with respect to the X-ray orientation), as well as the best scoring within the set of docking poses.

Moreover, it is noticeable (Figure 1c) that, in general, large NOE R-factors are associated with large ligand RMSDs, indicating that R-factors themselves are useful to identify 3D models agreeing with the crystallographic data. It is also worth noting that, for this protein–ligand system (and also for the two complexes discussed below), few docking poses showed relatively high RMSD values (3 to 5 Å) yet having very good agreement with the STD NMR experimental data (NOE R-factor <0.3; poses shown in the ESI Figure S1a). This suggests that the use of a static 3D model of the protein for the docking calculations fails to predict properly the inherent dynamics of the binding pocket and reflects on the important fact that, for small molecules, a binding pocket could accommodate other orientations of the ligand different to that observed in X-ray crystallography, still being compatible with the experimental NMR data in solution. For that reason, we explore the dynamics of the system by testing *RedMat* on molecular dynamics simulations.

#### Validation of Dynamic 3D Models

We then assessed the strength of the reduced matrix approach to monitor ligand reorientations, ligand association/dissociation events, and protein binding pocket side chain adaptation to different ligand orientations. To do so, we applied *RedMat* to the analysis of funnel metadynamics (funnel-MD) simulations.^38^

Funnel-MD relies on defining a funnel-shaped conformational space where the broad and narrow ends correspond, respectively, to the conformational space of the protein (ligand-bound state) and the solvent (ligand-unbound state) that the ligand is allowed to sample. Briefly explained, this is achieved by applying a repulsive potential outside the funnel space and, hence, promoting ligand association and dissociation events in a short simulation time.

Figure 1d shows the superposition of the X-ray crystal structure (yellow sticks) and three frames from the funnel-MD (sticks in magenta) of the *Rg*NanH-GH33–2,7-anhydro-Neu5Ac complex. Along the 400 ns funnel-MD a total of 14 dissociation/association events were observed. Notably, between 340 and 360 ns, the ligand adopted an orientation in the binding site analogous to the X-ray one, which was reflected as a region of low NOE R-factors (below 0.3, Figure 1e; ESI Figure S2) and ligand RMSD (with respect to the ligand X-ray orientation; Figure 1f; ESI Figure S2 and Movie S1).

Hence, we can reliably conclude that the combination of funnel-MD with *RedMat* represents a novel and unique computational approach not only to validate 3D models of low-affinity protein–ligand complexes but to generate and monitor them and to efficiently identify the ensemble of structures compatible with the experimental STD NMR data, in a relatively short time (ca. 200 ns of funnel-MD trajectory per day for a system of about 50,000 atoms). Further, we also performed classical MD simulations for this complex. The *RedMat* analysis of the 100 ns trajectory showed that the model was in very good agreement with the experimental data for the first 45 ns, with an average NOE R-factor of 0.10 (standard deviation of 0.01). However, at this point, the ligand underwent a conformational change that resulted in a pose/conformation with significantly poorer agreement with the experimental binding epitope. This may be due to the limitations of the force field parameters used. In this context, problems intrinsic to the force field parametrization can be overcome by introducing experimental restraints to reduce the conformational space that the system can explore, thus driving it toward configurations that best match the existing experimental data. Thus, we introduced some experimental restraints into the model based on information available from a previous DEEP-STD NMR study.^23^ These were restraints between protons H9/H9’/H8 of the ligand with Trp 698, proton H3a of the ligand with Ile 258, and protons of the ligand methyl group with the methyl groups of Val 502 and Ile 338. All restraints allowed each pair to have a distance range between 2 and 6 Å, based on NOE being detectable within these ranges. With these restraints, the ligand remained in the crystallographic binding pose/conformation for most of the MD trajectory (ESI Figure S3b), showing an extremely good agreement with the experimental binding epitope (except for the last 3 ns, due to a slight reorientation of the ligand), with an average NOE R-factor of 0.11 (standard deviation of 0.02) (ESI Figure S3c). This example further highlights the power of combining the latest advanced multifrequency STD NMR techniques with *RedMat* to generate either a 3D model or a dynamic ensemble of the complex showing agreement with the experimental data in solution.

### Bromodomain-Containing Protein 4 (BRD4) Bound to Two Different Pyridazine Ligands

**Figure 2:**
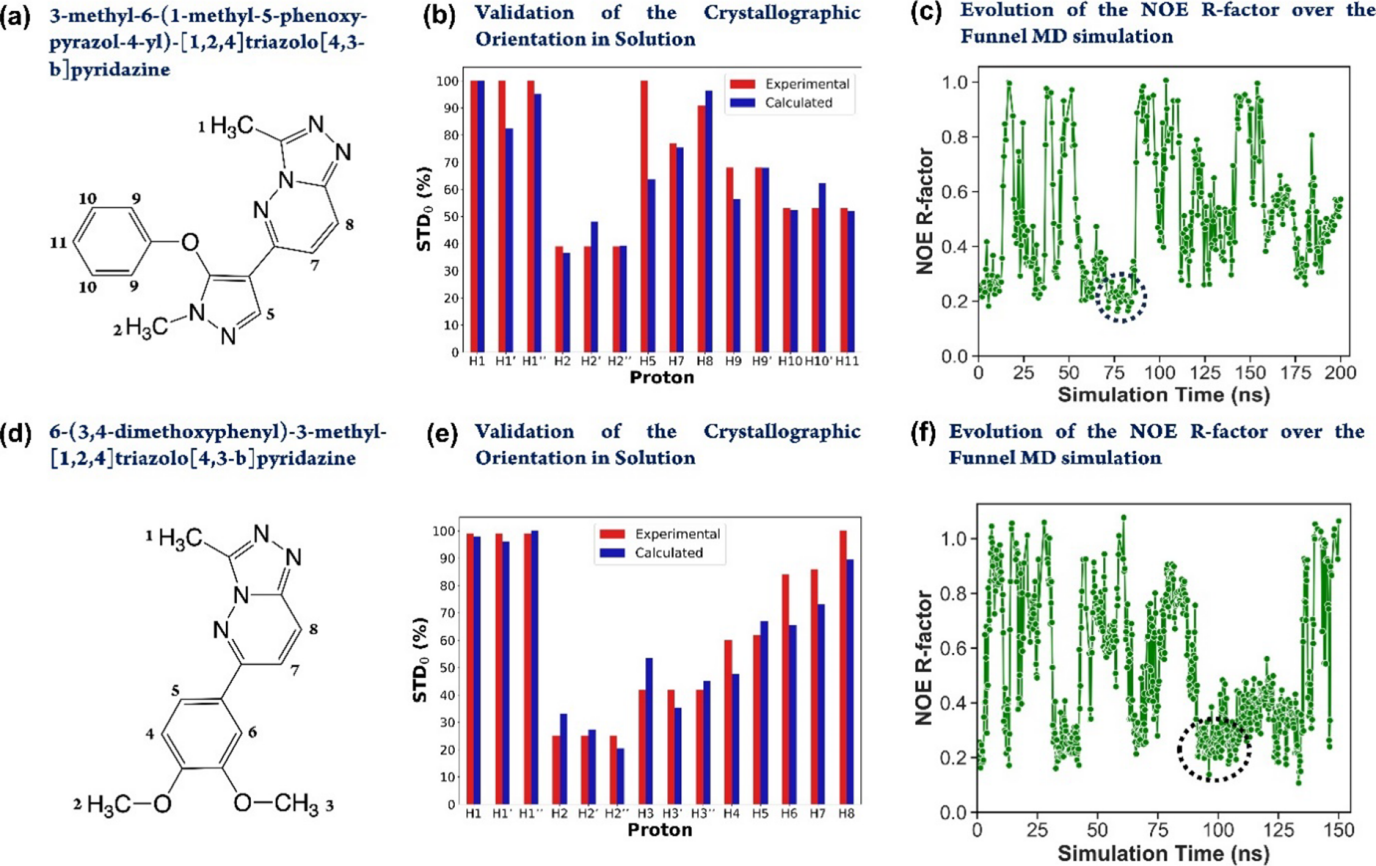
*RedMat* analysis of two triazolopyridazine ligands binding to BRD4 (PDB codes 5M3A (a–c) and 5M39 (d–f)). (a,d) 2D sketch of the structure of 3-methyl-6-(1-methyl-5-phenoxy-1H-pyrazol-4-yl)[1,2,4]triazolo[4,3-*b*]pyridazine (a, **Ligand 1**) and 6-(3,4-dimethoxyphenyl)-3-methyl[1,2,4]triazolo[4,3-*b*]pyridazine (d, **Ligand 2**). Ligand proton numberings are shown. (b,e) Comparison between the calculated (from the X-ray structure; blue bars) and experimental (red bars) relative STD_0_ factors (binding epitope mapping) of the non-exchangeable protons of **Ligand 1** (b) and **Ligand 2** (e) binding to BRD4. NOE R-factors of 0.16 and 0.12 were obtained (using a cutoff of 10 Å), respectively, showing a very good agreement between the crystal and solution-state structures of the complexes. (c–f) Evolution of the NOE R-factor over the funnel-MD simulation of **Ligand 1** (c, cutoff of 12 Å) and **Ligand 2** (f, cutoff of 10 Å) binding to BRD4. The segments of the trajectories where both ligands adopt an X-ray-type of orientation are highlighted with a dashed oval and correlate well with the lower ligand RMSD regions along the simulations (ESI, Figure S5).

Finally, we also assessed the application of *RedMat* to FBDD. To that aim, we applied our new approach to study the molecular recognition of two fragment molecules (i.e., of molecular weight below 300 Da; Figure 2a,d) to the protein receptor bromodomain-containing protein 4 (BRD4; PDB codes 5M3A and 5M39). The experimental STD NMR binding epitopes of both ligands were retrieved from the literature^39^ and compared with the *RedMat*-calculated epitopes (Figure 2b,e).

For *RedMat* calculations, we used a rotational correlation time of the protein of 10 ns, estimated with HYDRONMR.^37^ The protein and ligand concentrations were 45 μM and 1000 μM, respectively, according to the experimental conditions, as reported by Geist et al.^39^Figure 2b,e shows the comparison of the experimental (red bars) and theoretical (blue bars) epitopes of **Ligand 1** (2b) and **Ligand 2** (2e) obtained using *RedMat* for the X-ray structures 5M3A and 5M39. NOE R-factors of 0.16 and 0.12 were obtained (using a cutoff of 10 Å) for **Ligand 1** and **Ligand 2**, respectively, showing a very good agreement between the crystal and solution-state structures of the complexes. Further, similar to the 4X4A complex previously described, docking calculations also showed that, in general, (i) large NOE R-factors are associated with large ligand RMSDs and (ii) few docking poses showed relatively high RMSD values (3–5) while presenting an NOE R-factor below 0.3 (ESI Figures S1b,c and S4a,b).

Funnel-MD simulations allowed to generate and validate 3D complexes in very good agreement with experimental STD NMR data within short simulation times, as shown by the evolution of the NOE R-factor along the trajectories (Figure 2c,f). Importantly, both ligands adopt X-ray-like orientations along the funnel-MD, and these correspond to funnel-MD fragments presenting both lower NOE R-factor and ligand RMSD (Figure 2c,f; ESI Figures S4c,d, S5 and Movies S2 and S3) values.

In summary, these results demonstrate that *RedMat* is (i) robust and can provide accurate calculations, and (ii) in combination with funnel-MD simulations, the precision of *RedMat* calculation is greatly boosted.

This is an area of paramount importance in FBDD. Further, we suggest that other enhanced sampling MD methods like replica exchange^40^ and simulated annealing^41^ could be used.

It is worth noting that, when studying previously uncharacterized protein–ligand complexes, it is recommended to first assess ligand specificity. Thus, for those cases where the predicted binding epitope for static and dynamic 3D models do not reasonably match the experimental data (i.e., NOE R-factors over 0.3), the presence of nonspecific binding and/or multiple binding modes should be investigated via the use of a competitor ligand, by monitoring the binding epitope at increasing ligand concentrations^42^ or using paramagnetic probes.^31^

As a note of caution, since RedMat calculations rely on the available experimental STD NMR information, for ligands with very few protons and/or overlapping ^1^H peaks introducing uncertainty in the binding epitope, it is more likely to obtain both false-positive and false-negative results. False-positives meet NOE R-factor criteria but may not represent the correct ligand orientation, while false-negatives could show a correct orientation but lack STD NMR data for critical binding hotspots. Using RedMat alongside complementary methods such as site-directed mutagenesis is advised in such cases (see also the final note in the ESI).

We should finally note that two more protein–ligand complexes (PDB codes 6MSY and 6GH2) have also been subjected, with success, to *RedMat* analysis, and the results are shown and discussed in the ESI (Figures S6 and S7). In addition, CORCEMA-ST calculations for 6GH2 and the two BRD4 complexes are shown in the ESI and compared with RedMat results and the experimental epitopes (Figures S7a and S8).

## Conclusions

In conclusion, we have developed a reduced relaxation matrix theoretical approach that allows very fast validation of static and dynamic 3D models of low-affinity protein–ligand complexes against experimentally determined STD NMR binding epitopes. The high computational speed of the new algorithm allows efficient determination of theoretical binding epitopes from STD_0_ factors using the Cartesian coordinates of the 3D structure of the receptor–ligand complex, either in the form of a PDB structure or an MD trajectory. We have developed a practical implementation of this theoretical approach, in the form of a web application called *RedMat*, which has been tested on several protein–ligand complexes of biological or biotechnological relevance.

We show that *RedMat* is accurate (as evidenced by the low NOE R-factors obtained), robust, and, remarkably, very fast (on a time scale of seconds per complex on a desktop computer). The development of such a fast method to calculate theoretical STD_0_ factors to validate the 3D models of low-affinity protein–ligand complexes with experimental STD_0_ data in solution is of great interest to both academic research and the pharmaceutical industry. *RedMat* can easily be used in an efficient way to rapidly screen a large collection of protein–ligand complexes, obtained either from MD simulations or docking calculations, against experimental STD NMR data as part of drug discovery pipelines. Notably, we have further demonstrated that the application of *RedMat* analysis to funnel-MD trajectories constitutes a novel approach to significantly boost the generation and experimental STD NMR validation of 3D models of protein–ligand complexes.

We foresee that the efficient model validation framework that *RedMat* enables by combining molecular modeling and fast STD NMR binding epitope prediction can have a great impact in the fields of structural biology of low-affinity protein–ligand interactions and fragment-based drug discovery.

## Experimental Section

### Molecular Docking Calculations

The crystal structures of five protein–ligand complexes (PDB codes 4X4A, 5M3A, 5M39, 6MSY, and 6GH2) were used as starting coordinates and imported into the Maestro module of the Schrödinger software. First, protein structures were prepared using the Protein Preparation Wizard module.^43^ The PROPKA module was then employed to predict the protonation state of polar side chains at pH 7.5.^44^ The hydrogen-bonding network was optimized by sampling asparagine, glutamine, and histidine rotamers. The model was then minimized using the OPLS3 force field^45^ and a heavy-atom convergence threshold of 0.3 Å. Conformers of the ligands were generated in MacroModel^46^ using the MC/SD tool, and 100 different conformers were obtained. Clustering of conformers was carried out by heavy-atom RMSD to eliminate redundant poses, and 10 clusters were obtained. From each cluster, the lowest energy conformer was chosen based on the potential energy-OPLS3e term. A cubic docking grid was then generated centered on the position of the ligands of the X-ray structures with an inner and outer box length of 10 and 20 Å, respectively. Subsequently, docking calculations were performed with Glide^47^ using 10 conformers of each ligand. Flexible docking (i.e. protein residues are not allowed to move during the calculation) was carried out using the SP algorithm, and 100 poses per ligand conformer were obtained. Docking poses were then clustered by heavy-atom RMSD, and the poses closer to the centroid of each cluster were selected.

### MD Simulations

The initial atomic coordinates of each of the protein–ligand complexes were obtained from their crystallographic structures deposited in the Protein Data Bank: intramolecular trans-sialidase from *R. gnavus* in complex with 2,7-anhydro-Neu5Ac (4X4A), bromodomain 1 of bromodomain-containing protein 4 in complex with two pyridazine-like ligands (5M3A and 5M39), laminaribiose phosphorylase from *Paenibacillus sp.* in complex with α-Glc-1-phosphate (6GH2), and the broadly neutralizing anti-HIV-1 antibody 2G12 in complex with Man4 (6MSY). The results and discussion of complexes 6GH2 and 6MSY are included as Supporting Information (ESI Figures S6 and S7).

### Classical MD Simulations

Each system was parametrized using the AMBER ff14SB force field^48^ for the protein and GAFF^49^ (4X4A, 5M3A, 5M39, and 6GH2) or GLYCAM_06j-1^50^ (6MSY) force fields for the ligand. Ligand charges were determined using the antechamber software^51^ using the AM1-BCC level of theory. The systems were solvated with the TIP3P water model within a truncated octahedron bounding box buffered from the complex by 10 Å. Each system was neutralized with either Na+ or Cl– ions.

Conjugate gradient minimization was run with 20 kcal mol^–1^ Å^–2^ restraints on solute atoms, before repeating with no restraints. Each system was heated to 300 K over a period of 500 ps at constant volume, before equilibrating at constant pressure (1 atm) for a period of 2 ns. Production dynamics simulations were run for 100 ns each, with frames stored at 100 ps intervals (1000 frames). In all cases, periodic boundary conditions and the particle mesh Ewald method were applied. A Langevin thermostat with a collision frequency of 5 ps^–1^ and a Berendsen barostat with a relaxation time of 2 ps were used. The SHAKE algorithm was used to restrain all bonds involving hydrogen, allowing a time step of 2 fs. A cutoff of 8 Å was used for all nonbonded interactions.

In the case of 4X4A, we observed an excessive movement of the ligand within the protein-binding site. In order to improve the simulations, NOE-based restraints were applied between protons for which experimental STDs had been observed and protein side chains known from the X-ray crystal structure to be in close proximity.

The restraints applied a 20 kcal mol^–1^ Å^–2^ penalty for interatomic distances outside the 2–6 Å range, in agreement with the observed NOEs.

### Funnel Metadynamics Simulations

Funnel-MD simulations were carried out starting from the solvated systems (without any further equilibration), as described in previous sections, but using a truncated octahedron bounding box of 20 Å to improve the stability of the simulations. The coordinates of the funnel were generated using the funnel maker tutorial by Dominykas Lukauskis (https://github.com/dlukauskis/funnel_maker/tree), which is similarly described by Hedges et al.^52^ While the funnel-MD protocol was originally implemented by Limongelli et al.,^38^ the tutorial employed is based on the implementation described by Evans et al.^53^ and Saleh et al.^54^ This uses a single sigmoid function to make the funnel and improves the simulation performance by allowing the funnel to adapt to the protein movements.

Funnel-MD simulations were run using the OpenMM software (version 7.6).^55^ The funnel-MD protocol started with a short minimization, followed by 5 ns NVT equilibration and 5 ns of NPT equilibration with 5 kcal/mol of positional restraints applied to the ligand heavy atoms. The funnel-MD production run was performed at 300 K using an integration time step of 1 fs, an initial height of the Gaussian of 1.5 kJ/mol, and a friction coefficient to couple the system to the heat bath of 1 ps^–1^.

## Data Availability

The software resulting from this work, *RedMat*, is freely available for academic users upon request to the authors for granting access to our server-based application (see the ESI for a detailed user guide). The Web site is accessible via the following link http://redmat.iiq.us-csic.es/.

## Supporting Information Available

Association event between the 2,7-anhydro-Neu5Ac ligand and the *Rg*NanH-GH33 protein during a funnel-MD trajectory (MP4)
Association event between **Ligand 1** and the BRD4 protein during funnel-MD simulations (MP4)
Association event between **Ligand 2** and the BRD4 protein during funnel-MD simulations (MP4)
Experimental and theoretical STD_0_ values, a step-by-step user guide to perform *RedMat* calculations on our Web server, results showing association events during the funnel-MD simulations with convergence toward the X-ray orientation, and note on the potential limitations of the Reduced Relaxation Matrix Approach (PDF)
PDB files of the docking poses used for RedMat calculations (together with a spreadsheet containing docking scores and R-NOE values for each) (ZIP)

## Abbreviations

FBDD: fragment-based drug discovery
NMR: nuclear magnetic resonance
STD: saturation transfer difference
DEEP-STD: differential epitope mapping STD
