# Supplementary material for: Fast Quantitative Validation of 3D Models of Low-Affinity Protein–Ligand Complexes by STD NMR Spectroscopy
Source: J Med Chem. 2024 Jun 7;67(12):10025–34. doi: 10.1021/acs.jmedchem.4c00204 (PMC11215723; doi:10.1021/acs.jmedchem.4c00204)
Supplement: Supplementary file 4 — jm4c00204_si_004.pdf [file jm4c00204_si_004.pdf]

Supporting Information

Fast Quantitative Validation of 3D Models of Low-Affinity Protein-Ligand Complexes by STD NMR Spectroscopy

Ridvan Nepravishta*[a,c], Jonathan Ramírez-Cárdenas[b], Gabriel Rocha[b], Samuel Walpole[a], Thomas Hicks[a], Serena Monaco[a], Juan C. Muñoz-García*[b] and Jesús Angulo*[a,b]

[a] School of Pharmacy, University of East Anglia
Norwich Research Park, NR4 7TJ Norwich, UK

[b] Instituto de Investigaciones Químicas (IIQ-CSIC)
49 Américo Vespucio St. 41092, Sevilla, Spain
E-mail: juan.munioz@iiq.csic.es; j.angulo@iiq.csic.es

[c] Cancer Research Horizons, CRUK Scotland Institute
Garscube Estate, Switchback Road, Bearsden, Glasgow
G61 1BD, UK
E-mail: ridvan.nepravishta@cancer.org.uk

TABLE OF CONTENTS

Results S2

Human anti-HIV-1 antibody 2G12 – tetramannoside complex S5

Laminaribiose phosphorylase – α-glucopyronase-1-P complex S5

RedMat and Corcema-ST STD0(%) calculation for BRD4 complexes 5M3A and 5M39 S7

Raw data S8

RedMat step-by-step user guide S9

A final note about the potential limitations of the Reduced Relaxation Matrix Approach S13

REFERENCES S13

Results

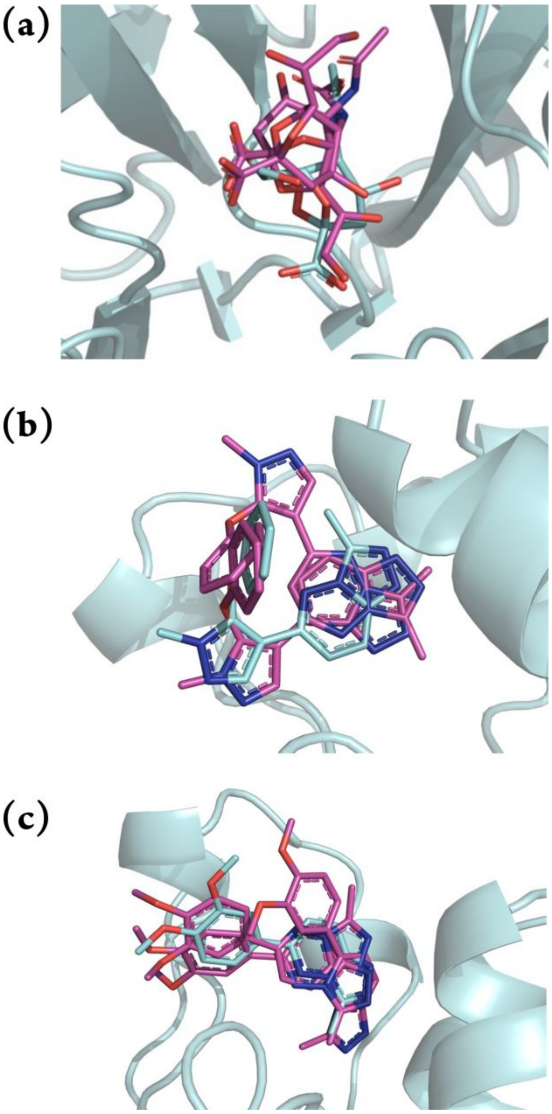

Figure S1. Superposition of the ligand poses obtained from docking calculations with low NOE-R factor and moderately high ligand RMSD (wrt the x-ray orientation) and the cristal structure. (**a**) Complex between the 2,7-anhydro Neu5Ac ligand and *Rg*NanH-GH33 (PDB code 4X4A). Docking poses within a ligand RMSD range of 3.2-4.2 and NOE-R factor range of 0.18-0.22 are shown as magenta sticks while the ligand crystallographic orientation is shown as cyan sticks. (**b-c**) Complexes between two pyridazine ligands (Ligand 1-(b), Ligand 2-(c)) and BRD4 (PDB codes 5M3A-(b) and 5M39-(c)). Docking poses within a ligand RMSD range of 2.0-4.0 and NOE-R factor range of 0.12-0.19 are shown as magenta sticks while the ligand crystallographic orientation is shown as cyan sticks.

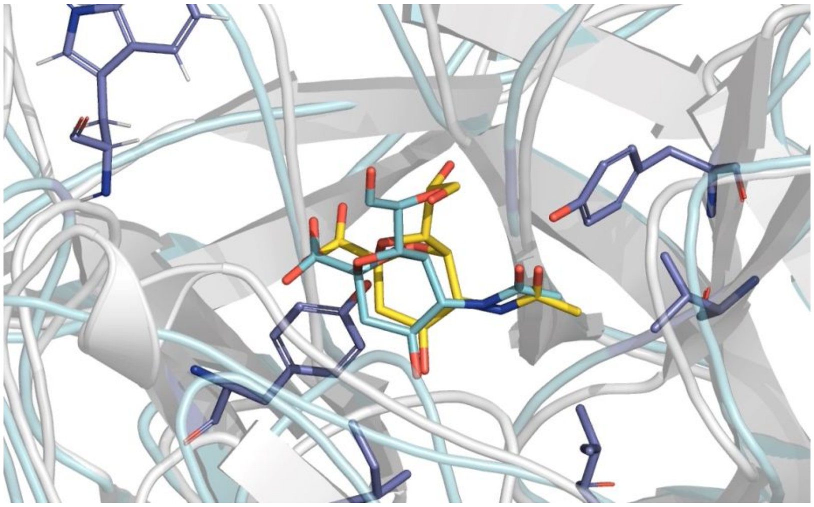

Figure S2. Snapshot of one of the association events between the 2,7-anhydro Neu5Ac ligand (shown as cyan sticks) and the *Rg*NanH-GH33 protein during a funnel-MD trajectory (see also video 1 provided as supporting information). Convergence towards the ligand crystallographic orientation (shown as yellow sticks) is observed, which corresponds to frames of low RMSD and NOE R-factor values (as shown in Fig. 1e,f in the main text).

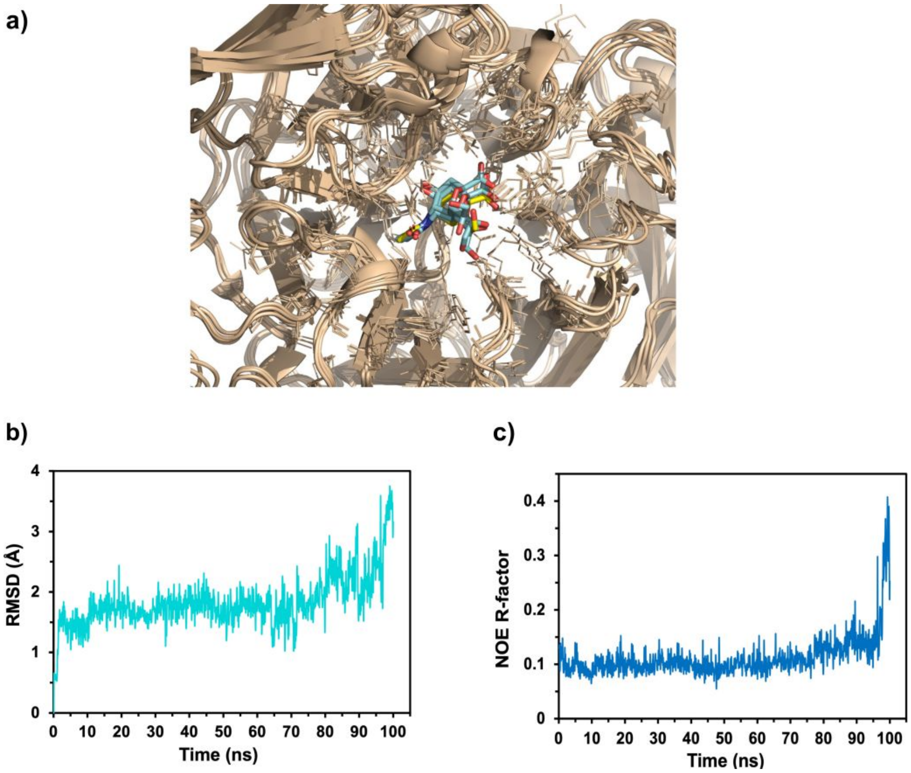

Figure S3. RedMat analysis of the 2,7-anhydro-Neu5Ac binding to RgNanH-GH33 (PDB 4X4A) during a 100-nanosecond classical MD simulation. (**a**) Superposition of 3 frames of the MD simulation (protein in wheat colored cartoon, ligand in cyan sticks) and the X-ray structure (ligand in yellow sticks) of the complex. The protein residues within 12 Å from the ligand, shown as wheat-colored lines, were included in the calculation of theoretical STD_0 values. (**b**) Evolution of the RMSD of 2,7 anhydro Neu5Ac (all atoms except the protons considered), with respect to the protein binding site (residues within 6 Å from the ligand considered), over the MD simulation. (**c**) Evolution of the NOE R-factor of 2,7 anhydro Neu5Ac over the MD simulation.

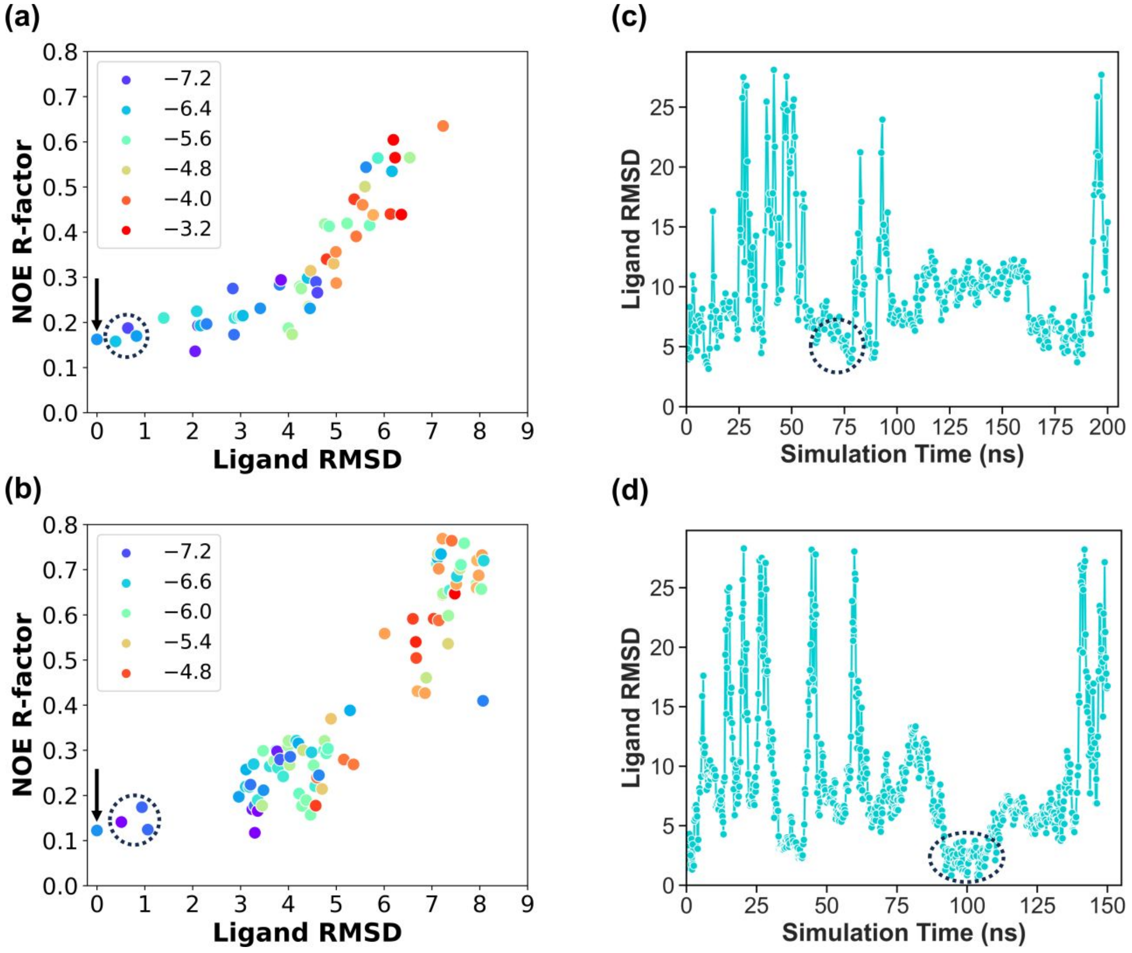

Figure S4. (a-b) 2D plot representing the NOE R-factor vs the ligand RMSD for the docking poses obtained for Ligand 1 (a) and Ligand 2 (b) binding to BRD4. The docking score of each pose is indicated by the color code shown in the legend. The data point corresponding to the X-ray structure is highlighted with an arrow and the docking poses resembling the crystallographic orientation are indicated with a dashed circle. **(c-d)** Evolution of the root mean squared deviation (RMSD) for Ligand 1 (c) and Ligand 2 (d) (all atoms except the protons considered) with respect to the protein binding site of BRD4 (residues within 6 Å from the ligand considered). The fragments of the trajectories where the ligands adopt a X-ray-type of orientation are highlighted with a dashed circle.

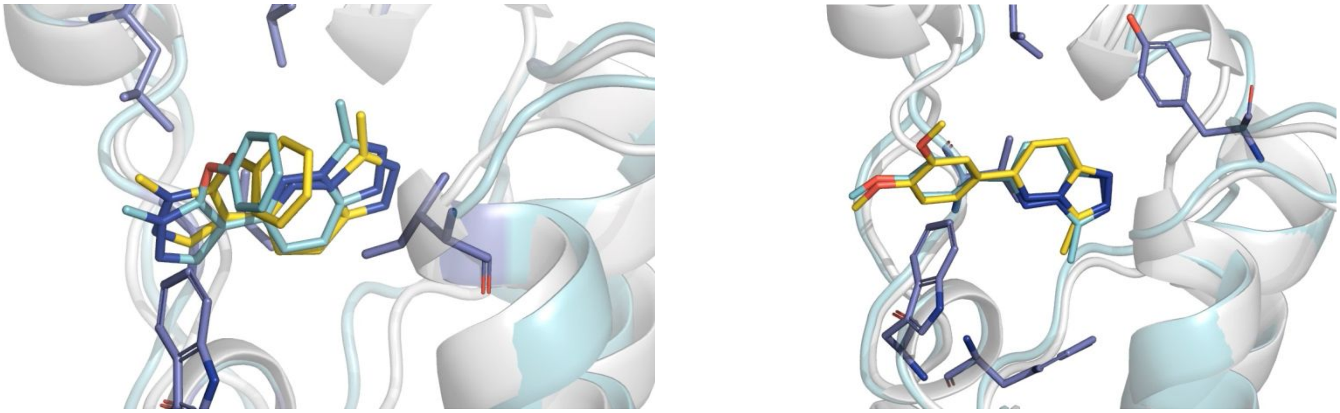

Figure S5. Snapshots of one of the association events between Ligand 1 (left) and Ligand 2 (right) (shown as cyan sticks) with the BRD4 protein during funnel-MD simulations (see also videos 2 and 3 provided as supporting information). Convergence towards the ligand crystallographic orientation (shown as yellow sticks) is observed for both ligands, this corresponding to frames of low RMSD and NOE R-factor values, as shown in Fig. 2c and 2f of the main text for Ligand 1 and Ligand 2, respectively.

Human anti-HIV-1 antibody 2G12 – tetramannoside complex.

We have also studied the human monoclonal antibody 2G12 in complex with a tetramannoside (PDB code: 6MSY), which is of therapeutic interest due to its role as a broadly neutralizing antibody against human immunodeficiency virus (HIV)[1]. For the RedMat calculation, we used a rotational correlation time of the protein of 43.1 ns, estimated with HYDRONMR[2], and a dissociation constant of 1000 μM. The ligand and protein concentrations were 8000 μM and 25 μM, respectively, according to the experimental conditions.[3] Figure S6 shows a superposition of the the X-ray crystal structure and 3 MD trajectory frames of the 2G12-tetramannoside complex (panel b), and the comparison of experimental STD_0 values (blue bars) with those calculated using RedMat (red bars; panel a). An NOE R-factor of 0.18, using a cutoff of 18 Å, was obtained for the X-ray structure, indicating a good fit between the experimental and theoretical STD_0 values. We next switched to the dynamics mode by analyzing a 100 ns unbiased MD simulation of the complex. The RedMat analysis of the resulting MD trajectory showed an average NOE R-factor of 0.18 (with a low standard deviation of 0.02), indicating that the complex remained relatively stable throughout the simulation generating a dynamic ensemble fully compatible with the experimental STD NMR data (Fig. S6d).

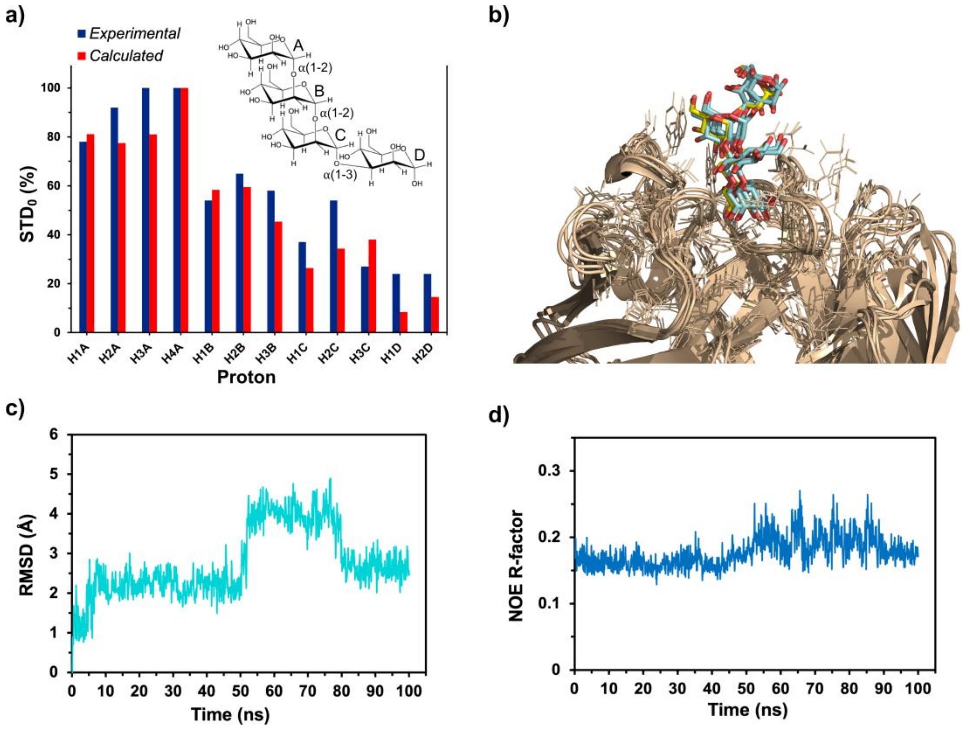

Figure S6. a) Comparison between the calculated (from the X-ray structure; red bars) and experimental (blue bars) relative STD_0 factors (binding epitope mapping) of the non-exchangeable protons of a tetramannoside bound to the anti-HIV 2G12 monoclonal antibody (PDB 6MSY). A NOE R-factor of 0.18 was obtained using a cutoff of 18 Å, hence showing a very good agreement between the crystal and solution state structures of the complex. A 2D sketch of the ligand is also shown, with the labels associated with each sugar ring shown next to it. **b)** Superposition of 3 frames of the MD simulation (protein in wheat-colored cartoon, ligand in cyan sticks) and the X-ray structure (protein in wheat colored cartoon, ligand in orange sticks) of the complex. The protein residues within 12 Å from the ligand, shown as wheat-colored lines, were included in the calculation of the theoretical STD_0 values. **c)** Evolution of the root mean squared deviation (RMSD) of the tetramannoside ligand (all atoms considered except the protons) with respect to the protein binding site (residues within 6 Å from the ligand). **d)** Evolution of the NOE R-factor of the tetramannoside ligand over the 100 ns MD simulation.

It is noteworthy that a significant increase in ligand RMSD with respect to the binding site occurs between 52 and 77 ns (Fig. S6c), due to a conformational change of the ligand α(1-3) glycosidic bond. This leads to a reorientation of the mannose residue at the reducing end (D), which experimentally shows very small STD_0 values as it is the most solvent exposed ring of the tetramannoside ligand (Figure S6a). For this reason, such a ligand conformational change leads to only a very small increase in the NOE R-factor (Fig. S6d). This highlights that significant changes in ligand RMSD along an MD simulation do not necessarily correlate with significant changes in the agreement with the experimental binding epitope mapping when conformational changes affect regions of the ligand with low experimental STD values, and emphasizes the great potential of combining MD simulations and RedMat analysis to generate and validate 3D ensemble structures of flexible protein-ligand complexes that best represent the experimental data in solution.

Laminaribiose phosphorylase – α-glucopyronase-1-P complex.

Finally, we also studied the complex of the GH94 laminaribiose phosphorylase enzyme from *Paenibacillus sp.* YM1 (PsLBP) with the ligand α-glucopyranose-1-phosphate (PDB code: 6GH2).[4] The PsLBP reverse reaction (synthesis of glycosidic linkages) is of high importance because it can be used as an alternative way of enzymatic glycosylation using sugar-1-phosphates as donor substrates.[5,6] For the RedMat calculation, we used a protein rotational correlation time of 68.5 ns, estimated with HYDRONMR[2] (GH94 has a molecular weight of ≈ 102 kDa), and a dissociation constant of 2000 μM. The concentrations of ligand and protein were 5000 μM and 50 μM, respectively, according to the experimental conditions.[4]

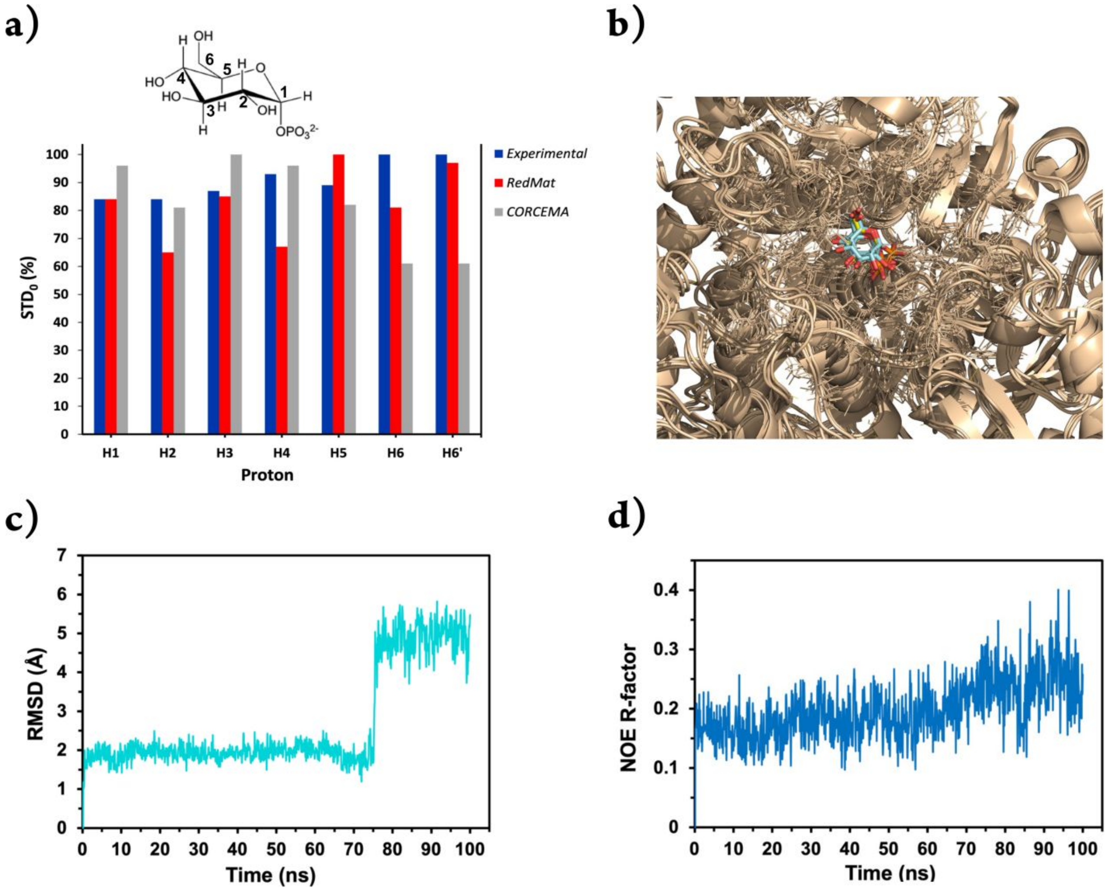

Figure S7. a) Comparison between the calculated (for the X-ray structure 6GH2; RedMat - red bars, CORCEMA-ST – gray bars) and experimental (blue bars) relative STD_0 factors (binding epitope mapping) of the non-exchangeable protons of α-Glc-1-phosphate bound to *PsLBP*. A NOE R-factor of 0.16 and 0.24 was obtained from RedMat and CORCEMA-ST theoretical calculations, respectively, using a cutoff of 15 Å. A 2D sketch of the ligand is also shown, with the labels associated with each sugar ring shown next to it. **b)** Superposition of 3 frames of the MD simulation (protein in wheat colored cartoon, ligand in cyan sticks) and the X-ray structure (protein in wheat colored cartoon, ligand in orange sticks) of the complex. The protein residues within 12 Å from the ligand, shown as wheat-colored lines, were included in the calculation of theoretical STD_0 values. **c)** Evolution of the root mean squared deviation (RMSD) of the tetramannoside ligand (all atoms except the protons considered) with respect to the protein binding site (residues within 6 Å from the ligand). **d)** Evolution of the NOE R-factor of α-Glc-1-phosphate over the 100 ns MD simulation.

Figure S7 shows the superposition of the X-ray crystal structure and three MD trajectory frames of the *a*-Glc-1-phosphate-GH94 laminaribiose phosphorylase complex (panel b). We compared also the results obtained using both RedMat and CORCEMA-ST (red and grey bars, respectively; panel a). The NOE R-factor of the X-ray model was 0.16 and 0.24 as calculated by RedMat and CORCEMA-ST, respectively (using a cutoff of 15 Å), hence in good agreement with the STD NMR epitope mapping. With regards to the MD simulation, the ligand remained stable in the binding site for the first 75 ns (Fig. S7c), showing an average NOE R-factor of 0.18 (standard deviation of 0.04) (Fig. S7d).

RedMat and Corcema-ST STD_0(%) calculation for BRD4 complexes 5M3A and 5M39 .

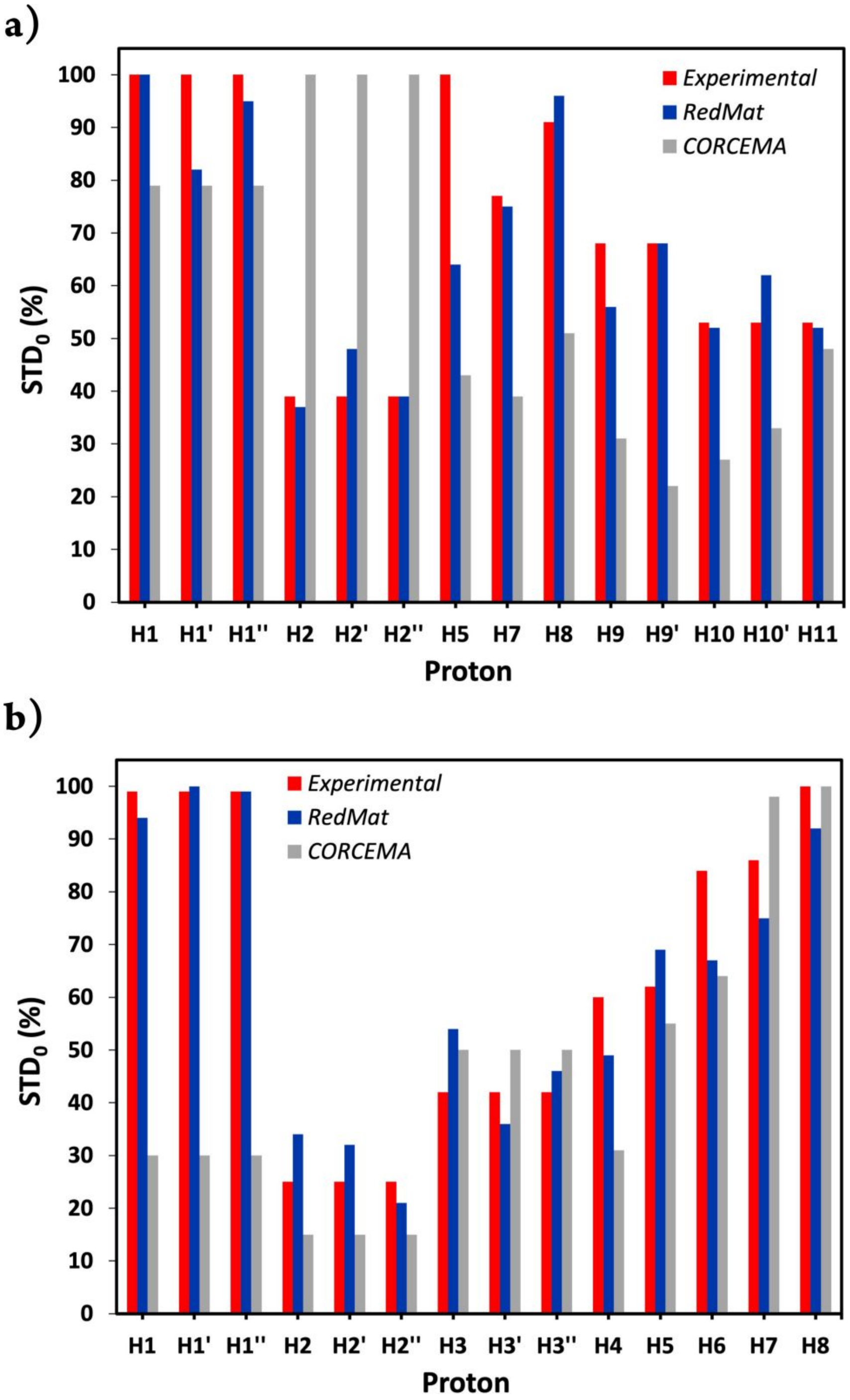

Figure S8. Comparison between calculated (RedMat - red bars, CORCEMA-ST – gray bars) and experimental (blue bars) relative STD_0 factors (binding epitope mapping) of the non-exchangeable protons of Ligand 1 (a) and Ligand 2 (b) binding to BRD4 (PDB codes 5M3A and 5M39, respectively). For Ligand 1 (a) NOE R-factors of 0.17 and 0.62 were obtained from RedMat and CORCEMA-ST calculations, respectively. For Ligand 2 (b) NOE R factors of 0.13 and 0.65 were obtained from RedMat and CORCEMA-ST calculations, respectively. In all cases, a distance cutoff of 10 Å was used for the calculations.

For both BRD4 complexes, CORCEMA-ST yields poor results compared to experiments, being greatly overcome by RedMat performance. However, we should note that only the experimental binding epitope was reported for these two complexes but not the experimental STD NMR build-up curves, hence hindering the direct comparison with CORCEMA-ST simulated curves. In addition, we should emphasise that CORCEMA-ST relies on calculating the whole build-up of saturation transfer onto the ligand protons along all saturation times and, hence, it is highly dependent on several parameters (kinetics of exchange, global and local correlation times, proton longitudinal relaxation times, etc.) which can introduce bias. In that sense, our results indicate that, when CORCEMA-ST shows great performance (very low NOE R-factor), RedMat reproduces very well the predicted outcomes (ESI Fig. S7). On the other hand, when CORCEMA-ST results are not very “reliable” (i.e. either the NOE R-factor is above 0.3 or the whole experimental build-up curves are not available for direct comparison with

CORCEMA build-up curves), RedMat outperforms CORCEMA-ST (ESI Fig. S8). However, in the latter case we should be cautious and carry out a detailed comparison with the experimental build-up curves.

Raw Data

Table S1. Experimental and theoretical (RedMat; cutoff distance of 18 Å) STD_0 values of a tetramannoside bound to anti-HIV 2G12 monoclonal antibody (PDB code: 6MSY).

Proton	Experimental STD_0	Theoretical STD_0
Man1 H1	78	100
Man1 H2	92	91
Man1 H3	100	59
Man1 H4	100	100
Man2 H1	54	53
Man2 H2	65	82
Man2 H3	58	47
Man3 H1	37	18
Man3 H2	54	27
Man3 H3	27	29
Man4 H1	24	13
Man4 H2	24	17

Table S2. Experimental and theoretical (RedMat; cutoff distance of 12 Å) STD_0 values of 2,7anhydroNeu5Ac bound to RgNanHGH33 (PDB code: 4X4A).

Proton	Experimental STD_0	Theoretical STD_0
H32	78	66
H33	78	70
H4	83	76
H5	76	70
H6	77	63
H7	76	55
H8	46	39
H92	36	23
H93	36	29
H111	100	94
H112	100	100
H113	100	99

Table S3. Experimental and theoretical (RedMat; cutoff distance of 15 Å) STD_0 values of α-glucopyranose-1-phosphate bound to GH94 laminaribiose phosphorylase (PDB code: 6GH2).

Proton	Experimental STD_0	Calculated STD_0
H1	84	84
H2	84	65
H3	87	85
H4	93	67
H5	89	100
H62	100	81
H63	100	97

RedMat step-by-step user guide

1. Sign up / Log in

On a web browser, go to the URL http://redmat.iiq.us-csic.es/ and sign up (Fig. S7). The RedMat administrator will grant access to you. Please, note that we are currently working on the migration of RedMat to a different website domain, therefore, before signing up, we encourage users to contact the corresponding author for further details.

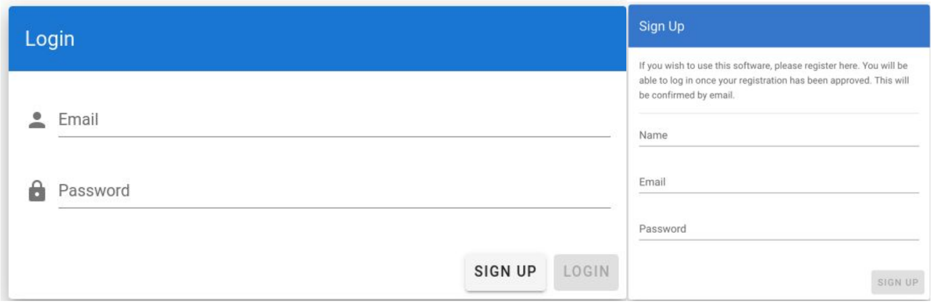

Fig. S9. Login (left) and sign up (right) windows of the RedMat web server.

2. Start calculation / Upload calculation results

Once logged in, you can choose from either uploading previous RedMat results (*.json* file required) or starting a new calculation (Fig. S8).

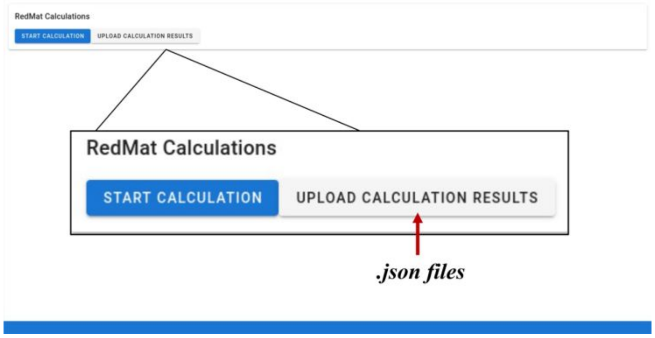

Fig. S10. Calculation options upon login onto the RedMat server.

3. Choose between Docking or Molecular Dynamics calculations

When clicking on "Start Calculation" a new window is opened where you can choose between Docking and Molecular Dynamics calculations (Fig. S9).

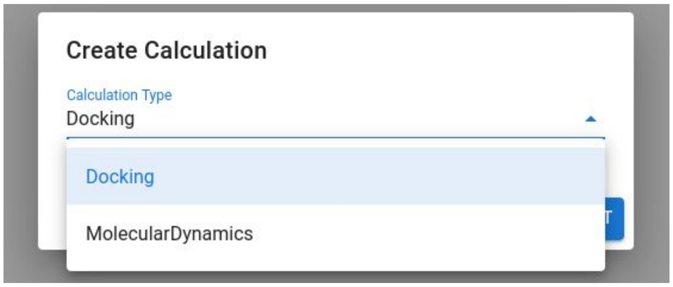

Fig. S11. RedMat window showing the two types of calculations available.

4. Define System - Upload files

Docking Calculations

Upload separate PDB files for the protein and ligand(s) (Fig. S11). Note that both the residue atoms and residue numbers in ligand and protein PDB files must be different.

Please, follow the recommendations detailed below:

(i) The AmberTools module *pdb4amber* can be employed to renumber the protein PDB file to start from atom number and residue number 1 (*e.g. pdb4amber -i inputfilename.pdb -o outputfilename.pdb*). The atom and residue numbers of the ligand PDB file should be manually

adjusted based on the last atom and residue numbers of the protein. If any atom or residue number of protein and ligand overlap an error will pop up. To easily adjust atom and residue numbers we recommend using pdb-tools (https://wenmr.science.uu.nl/pdbtools/).

(ii) For simplicity, we advise to remove all exchangeable ligand protons (*e.g.* OH, NH, SH) from the PDB file. Also, note that all the proton(s) for which no experimental STD_0 values are input are not considered in the calculation and, hence, they are not shown in the results window.

(iii) When preparing the ligand(s) PDB file, please be aware that some molecular visualization software (*e.g.* Maestro[7]) include the formal charge of the atom in the element column (last column) of the PDB file. For RedMat to read this file, please remove any numbers and symbols in this column apart from the element character.

(iv) Tens of ligand docking poses can be uploaded together in the same ligand PDB file to perform the RedMat calculation for all of them on a single run. To do so, the coordinates of each ligand pose in the PDB file must appear between the starting and ending MODEL (*MODEL_NUMBER)* and ENDMDL lines, respectively.

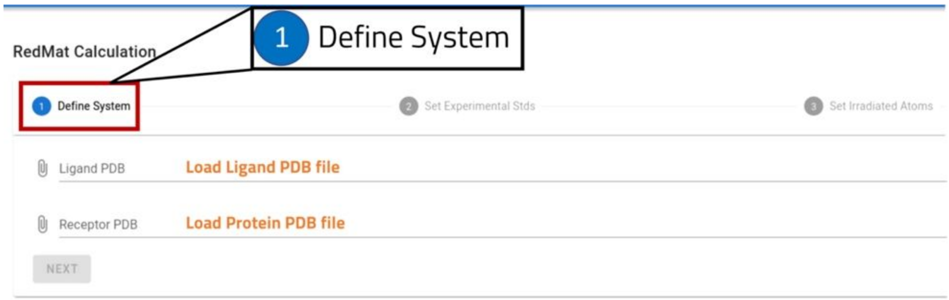

Fig. S12. File loading menu in the RedMat docking mode.

Molecular Dynamics Calculations

Same as in Fig. 11a, but two additional files must be uploaded (Fig. S12): (i) An AMBER topology file of the protein-ligand complex (*.prmtop* extension), (ii) A trajectory file of the protein-ligand complex (*i.e.* no water and no ions) in standard 10-column AMBER format (*.mdcrd* extension).

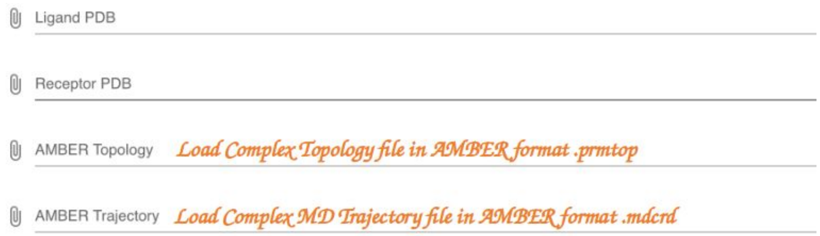

Fig. S13. File loading menu in the RedMat Molecular Dynamics mode.

5. Set Relative Experimental STD Initial Slopes (STD_0)

In this step, the relative (in %) experimental initial slopes (STD_0) for each proton of the ligand for which the STD factor could be determined (*i.e.* STD binding epitope) have to be manually set (Fig. S13).

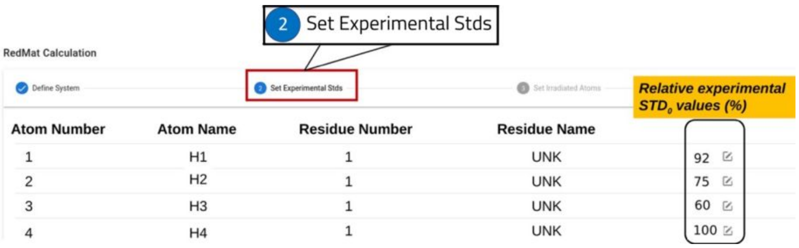

Fig. S14. RedMat menu to set the relative experimental STD_0 values (in %).

6. Set Irradiated Protons

Here, set the protein saturation frequency employed in your experiments. Three options are allowed: Methyl protons (irradiation around 0.5 ppm), aromatic protons (irradiation around 7.2 ppm), and a SHIFTX (http://www.shiftx2.ca/) csv file containing the residues of your protein that are predicted to be irradiated at the experimental irradiation frequency (Fig. S14). For best RedMat performance, we advise to use the SHIFTX option.

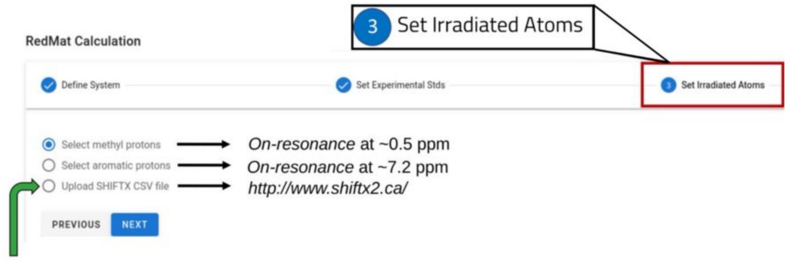

Residue number	Residue name	Atom name	Predicted Chemical Shift
11	L	HD1	0.674
11	L	HD2	0.796
14	I	HD1	0.617
14	I	HG2	0.680
14	I	HG13	0.854
18	T	HG2	0.984
20	I	HD1	0.654
20	I	HG2	0.413
20	I	HG13	0.867

Fig. S15. Menu to select the experimental irradiation frequency employed. Three options are allowed: Methyl protons (irradiation around 0.5 ppm), aromatic protons (irradiation around 7.2 ppm), and a SHIFTX csv file containing the residues of your protein that are predicted to be irradiated at the experimental irradiation frequency (the format of this file in the inset table).

7. Set Parameters

The most important parameters to set here are the Complex Correlation Time (in ps), which can be calculated with HydroNMR,[2] and the cutoff distance (in Angstrom), which represents the distance around the ligand for which to consider the directly irradiated and indirectly irradiated protein protons during the RedMat calculation. We advise RedMat users to screen for a range of cutoff values, typically from 10 to 14 Å, and select the results in best agreement with the experimental STD NMR binding epitope. Regarding the spectrometer frequency, ligand and receptor concentrations, and complex dissociation constant parameters, it should be noted that they have minimum influence on the results (Fig. S15).

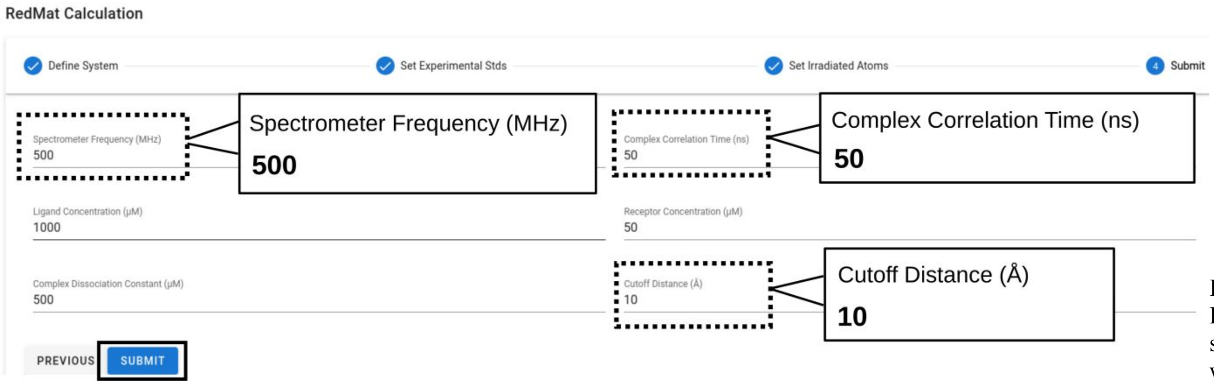

Fig. S16. RedMat submission window where some parameters can be set by the user.

8. Results window

The results window shows the average NOE R-factor for the whole ligand and for each individual proton. In the RedMat Docking mode, if more than one ligand docking pose/model is contained in the input ligand PDB file, the average NOE R-factor for each model is shown. In the RedMat MD mode, plots showing the evolution of the average NOE R-factor of the whole ligand (Fig. S16) and of each individual proton against each frame of the MD trajectory are also outputted.

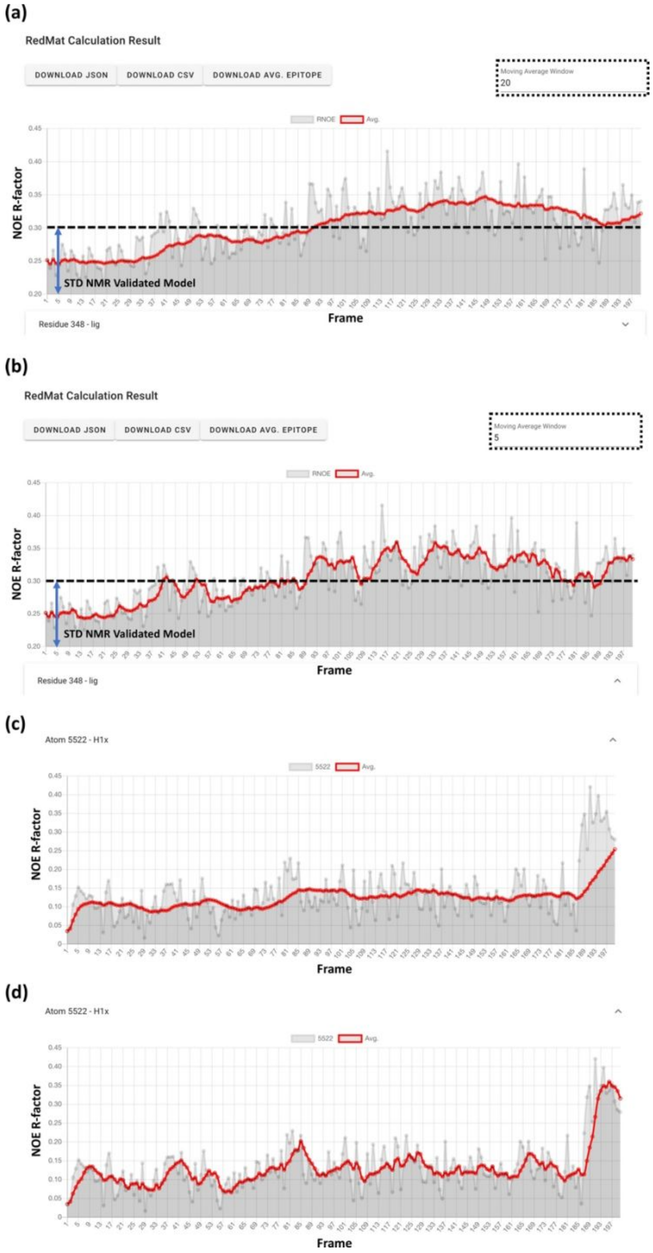

Fig. S17. RedMat results window for the analysis on an MD trajectory. (a-b) The evolution of the NOE R-factor of the ligand (grey line and dots) and the moving average NOE R-factor (red line and dots) over the last 20 (a) and 5 (b) frames are plotted in the output. The moving average window (upper right corner, dashed rectangle) can be set by the user. The dashed line drawn at a NOE R-factor of 0.30 indicates an upper limit for considering a 3D protein-ligand model to be in good agreement with the experimental ligand binding epitope by STD NMR initial slopes. (c-d) Evolution of the NOE R-factor of an individual ligand proton (grey line and dots) and the moving average NOE R-factor (red line and dots) using a window of 20 (c) and 5 (d) frames.

A final note about the potential limitations of the Reduced Relaxation Matrix Approach

The relaxation matrix, although essential for NOE calculations, comes with some well-known limitations. To obtain the relaxation matrix, the isotropic tumbling of the protein with a single correlation time is assumed.[8–10] This approximation might limit the accuracy of the structures during refinement steps. On the other hand, the inconsistency between predicted and experimental NOE might be due to simple motional averaging. For this reason, it is sometimes necessary to obtain a relaxation matrix from an ensemble of structures.[11] Moreover, due to previously mentioned approximations, the relaxation matrix might not be able to define a global minimum of the conformations of a ligand-protein system. In these cases, repeated Simulated Annealing and system convergence criteria should be considered together with relaxation matrix calculations. Similarly, simulated annealing, long MD simulations or replica exchange techniques should be considered for those systems where an agreement between experimental and calculated NOEs is not reached in the starting structure. Finally, in the

presence of few protons or in case of peak overlapping of ligand protons, the relaxation matrix calculations might be useful qualitatively to evaluate the protein-ligand structure, making these cases still very challenging.

REFERENCES

(1) Calarese, D. A.; Lee, H.-K.; Huang, C.-Y.; Best, M. D.; Astronomo, R. D.; Stanfield, R. L.; Katinger, H.; Burton, D. R.; Wong, C.-H.; Wilson, I. A. Dissection of the Carbohydrate Specificity of the Broadly Neutralizing Anti-HIV-1 Antibody 2G12. *Proc. Natl. Acad. Sci.* **2005**, *102* (38), 13372–13377. https://doi.org/10.1073/pnas.0505763102.

(2) l. Torre, J. G. de; Huertas, M. L.; Carrasco, B. HYDRONMR: Prediction of NMR Relaxation of Globular Proteins from Atomic-Level Structures and Hydrodynamic Calculations. *J Magn Reson* **2000**, *147* (1), 138–146.

(3) Enríquez-Navas, P. M.; Marradi, M.; Padro, D.; Angulo, J.; Penadés, S. A Solution NMR Study of the Interactions of Oligomannosides and the Anti-HIV-1 2G12 Antibody Reveals Distinct Binding Modes for Branched Ligands*. *Chem. – A Eur. J.* **2011**, *17* (5), 1547–1560. https://doi.org/https://doi.org/10.1002/chem.201002519.

(4) Kuhaudomlarp, S.; Walpole, S.; Stevenson, C. E. M.; Nepogodiev, S. A.; Lawson, D. M.; Angulo, J.; Field, R. A. Unravelling the Specificity of Laminaribiose Phosphorylase from Paenibacillus Sp. YM-1 towards Donor Substrates Glucose/Mannose 1-Phosphate by Using X-Ray Crystallography and Saturation Transfer Difference NMR Spectroscopy. *ChemBioChem* **2019**, *20* (2), 181–192. https://doi.org/https://doi.org/10.1002/cbic.201800260.

(5) Martínez, J. D.; Infantino, A. S.; Valverde, P.; Diercks, T.; Delgado, S.; Reichardt, N.-C.; Ardá, A.; Cañada, F. J.; Oscarson, S.; Jiménez-Barbero, J. The Interaction of Fluorinated Glycomimetics with DC-SIGN: Multiple Binding Modes Disentangled by the Combination of NMR Methods and MD Simulations. *Pharmaceuticals* **2020**, *13* (8), 179. https://doi.org/10.3390/ph13080179.

(6) KITAOKA, M.; MATSUOKA, Y.; MORI, K.; NISHIMOTO, M.; HAYASHI, K. Characterization of a Bacterial Laminaribiose Phosphorylase. *Biosci. Biotechnol. Biochem.* **2012**, *76* (2), 343–348. https://doi.org/10.1271/bbb.110772.

(7) Schrödinger Release 2023-1: Maestro Schrödinger LLC New York. Maestro Schrödinger, LLC, New York. Schrödinger Release 2023-1: Maestro, Schrödinger, LLC, New York, NY, 2021. 2021.

(8) Macura, S.; Ernst, R. R. Elucidation of Cross Relaxation in Liquids by Two-Dimensional N.M.R. Spectroscopy. *Mol. Phys.* **1980**, *41* (1), 95–117. https://doi.org/10.1080/00268978000102601.

(9) Ernst, R. R.; Bodenhausen, G.; Wokaun, A. Principles of Nuclear Magnetic Resonance in One and Two Dimensions. In *Magnetic Resonance Imaging*; Oxford University Press, 1988; Vol. 6, p 349. https://doi.org/10.1002/mrm.1910070215.

(10) Keepers, J. W.; James, T. L. A Theoretical Study of Distance Determinations from NMR. Two-Dimensional Nuclear Overhauser Effect Spectra. *J. Magn. Reson.* **1984**, *57* (3), 404–426. https://doi.org/https://doi.org/10.1016/0022-2364(84)90257-9.

(11) Bonvin, A. M.; Boelens, R.; Kaptein, R. Time- and Ensemble-Averaged Direct NOE Restraints. *J. Biomol. NMR* **1994**, *4* (1), 143–149. https://doi.org/10.1007/BF00178343.
